# Revisiting predictive biomarkers of musculoskeletal injury in thoroughbred racehorses: longitudinal study in polish population

**DOI:** 10.1186/s12917-019-1799-7

**Published:** 2019-02-26

**Authors:** Agnieszka J. Turlo, Anna Cywinska, David D. Frisbie

**Affiliations:** 1 Department of Pathology and Veterinary Diagnostics, Warsaw University of Life Sciences, ul. Nowoursynowska 159c, 02-776 Warsaw, Poland; 2 Gail Holmes Equine Orthopaedic Research Center, Colorado State University, 300 West Drake Road, Fort Collins, CO 80523 USA

**Keywords:** Racehorse, Serum biomarkers, Injury prediction, Musculoskeletal injury

## Abstract

**Background:**

High prevalence of musculoskeletal disorders in racehorses and its impact on horse welfare and racing economics call for improved measures of injury diagnosis and prevention. Serum biomarkers of bone and cartilage metabolism have previously shown promise in prediction of musculoskeletal injuries in horses. This study aimed to re-evaluate usability of the predictive serum biomarkers identified in North American Thoroughbred racehorses in a geographically distinct group of Polish Thoroughbreds.

**Results:**

Serum concentrations of bone and cartilage biomarkers: osteocalcin, c-terminal telopeptide of type I collagen, total glycosaminoglycans (GAG), chondroitin sulfate epitope and c-propeptide of type II procollagen (CPII) were evaluated in the beginning and the next 3 months of one racing season in a cohort of twenty-six 2-year-old Polish racehorses. Exit criteria were diagnosis of musculoskeletal injury, leading to > 5 days off training (*n* = 8), or completion of 3 study months with no training interruptions (*n* = 18). Normalized results and matching archival data from 35 2-year-old North American racehorses was used for logistic regression analysis to identify universal predictors of injury. Mean GAG and CPII levels were lower in injured group comparing to control, which is consistent with previous findings in racehorses. These biomarkers were also identified as predictors of injury in the mixed population model. Population origin had no significant effect on predictive value of evaluated biomarkers (Wald test *p* = 0.137). Decreased osteocalcin and increased c-terminal telopeptide of type I collagen levels in injured horses comparing to controls were specific for Polish population and signalized disruption in bone turnover homeostasis.

**Conclusions:**

Changes in serum GAG and CPII in racehorses at risk of injury appear to be similar across distinct populations while dynamics of serum bone marker is more population-specific.

**Electronic supplementary material:**

The online version of this article (10.1186/s12917-019-1799-7) contains supplementary material, which is available to authorized users.

## Background

Thoroughbred racehorses are enrolled in high-intensity training in their second year of life, before termination of skeletal growth [1]. Orthopaedic injuries are responsible for nearly 70% of the lost training days in Thoroughbred racing, with 2-year-olds failing to train more frequently and for longer time periods than older horses [2–5]. In all age groups, musculoskeletal injuries acquired during the high-intensity exercise are the major cause of on-track deaths as well as long-term interruptions in horse racing careers [2, 6–8].

Serious impact of stress injuries on horse welfare and horse racing economics encouraged the search for an effective diagnostic tool that would allow early identification of the individuals at risk. Biochemical serum markers can be particularly useful in monitoring of large groups of horses due to the quick and minimally invasive sample collection process. Research in blood biomarkers of equine musculoskeletal disease has shown promising results for diagnosis of congenital and acquired disorders [9–18] and prediction of injuries commonly acquired by racehorses [19–21]. Those studies evaluated products of bone and cartilage metabolism such as pro- and telopeptides of type I and type II collagen molecules, glycosaminoglycans, non-collagenous bone protein and osteocalcin. Bone and cartilage biomarkers evaluated in blood and synovial fluid can reflect changes in skeletal turnover related to pathologies as well as resulting from exercise-induced mechanical load [1, 22–24]. Therefore, training program and environment can be relevant factors affecting bone and cartilage metabolism in racehorses and should be accounted for in interpretation of biomarker analysis results.

Although musculoskeletal disorders are identified globally as the major health issue in racing Thoroughbreds, prevalence of specific injury types can vary between the countries or even individual training yards [2, 4]. The risk factors proposed as having impact on injury patterns in racehorse populations include training regimen (distance and speed) and racetrack characteristics (shape, length, surface condition) [4, 25–36]. Differences in commonly used track surfaces and training logistics are particularly evident between European and North American racing systems. Turf is a typical surface for European horse racetracks, while the large part of racing in North America is performed on dirt. Horses competing in different geographical locations are more prone to specific musculoskeletal pathologies which led to discrimination of European and North American Thoroughbreds as separate subjects of equine sports medicine [37, 38]. While these differences are widely recognized and addressed by epidemiological studies, attempts to include inter-population variability in equine biomarker research are lacking. The purpose of this study was: a) longitudinal evaluation of selected bone and cartilage biomarkers in the group of Polish Thoroughbred racehorses in their first training season; b) assessment of these biomarkers in a predictive model of injury, universal for Polish and North American racing Thoroughbreds. We hypothesized that different racing environments would lead to an altered pattern of biomarker changes in the Polish population compared to the results previously acquired in North American racehorses. However, we expected that the ability of some biomarkers to predict musculoskeletal injuries would prove to be independent of the population origin.

## Methods

### Animals

This was a longitudinal prospective study that involved 28 2-year-old Polish Thoroughbred racehorses in their first training season. According to the European directive EU/2010/63 and local law regulating animal experiments, there was no need for the approval of Ethical Committee for the described procedures, as the sample collections were performed as part of non-experimental clinical veterinary practice consented by the trainers and the owners of the horses. The study population was recruited from horses stabled and trained in one racing facility in Poland in the beginning of one racing season. Horses were trained and raced on turf track belonging to the racing facility. Three different trainers participated in the study with 12, 10 and 6 horses respectively. Horses entering the study were free of lameness, did not have history of musculoskeletal injury and did not show abnormalities in routine hematological examination (red blood cell count, hematocrit, total hemoglobin concentration, total white blood cell count, differential white blood cell count). Exit criteria were sustaining single musculoskeletal injury (injured group) or completion of three study months without showing clinical signs of injury (control group). Additionally, information on horse health was collected in the month following the last sample collection to ensure that horses classified as control did not sustain injury immediately after the study end, which may have affected analysis of the results. To be included as injured, the horse had to manifest clinical signs requiring veterinary attention and leading to loss of > 5 consecutive training days according to recommendation of a veterinary surgeon. Diagnosis was made by equine veterinarians cooperating with the racing stable and made available to the authors of the study. Injuries resulting from accidents (e.g., kick, fall) were not included in the study. All information regarding training regimen and injuries was collected during the monthly interview with horse trainers. Approximate training schedules implemented during the study period are presented in supplementary material (Additional file 1). Horses trained six days a week and each workout was preceded by a 10-min warm-up and followed by a 30-min cool-down in horse walker.

### Sample collection

Blood samples were collected in one-month intervals, starting with the baseline sample at the beginning of the study. Health and training information was acquired at the time of blood sample collection as well as horses were observed for cases of unreported lameness by research veterinary clinician collecting the samples. A total of four samples were collected from all horses over the three-month study period. Sampling took place between 6:00 and 7:00 AM after feeding and before daily exercise. Samples were collected by jugular venipuncture, using a blood collection system^1^ with a 20-gauge needle. Samples were centrifuged (4380 g for 5 min), serum was separated, divided into 1 ml aliquots into 1,5 ml Eppendorf tubes (3–4 aliquots/horse) and stored frozen at − 20 °C within 1 h of collection. After collection and during processing all samples were stored on ice.

### Biomarker analysis

The biomarker panel used in this study was selected based on the results of previously published equine biomarker studies [11–23, 39]. Five biomarkers that were demonstrated as useful in diagnosis and prognostication of musculoskeletal disease were: a) marker of bone formation, OC; b) markers of articular cartilage turnover, GAG and CS846; c) marker of type II collagen synthesis, CPII; d) marker of type I collagen breakdown, CTX I. These biomarkers are products of extracellular matrix metabolism in articular cartilage (CPII, GAG, CS846), bone (CTX I, OC) and tendon (CTX I) and provide information on the overall status of musculoskeletal system in the horse.

Biomarker analysis was undertaken at the Orthopaedic Research Laboratory, Colorado State University. The samples were transported from Poland on dry ice and remained frozen until analysis. All assays used for biomarker analysis were previously validated for use with equine serum [40, 41]. Osteocalcin level was measured by commercial ELISA kits^2^ in undiluted serum samples. Glycosaminoglycan concentration was analyzed by dimethyl-methylene blue assay in undiluted samples after papain digestion [42]. Chondroitin sulfate epitope and CPII were measured using commercial ELISA kits^3^ in samples diluted 1:5 and CTX I level was established with an ELISA^4^ in 1:4 dilution. All samples were analyzed in duplicates and measurements were repeated if intra-assay coefficient of variation (CV) exceeded 15%. The mean in-house intra-assay CV for respective test was: 12,0% (OC), 6,7% (GAG), 5,0% (CS846), 6,7% (CPII), 10,9% (CTX I).

### Historical data

To re-evaluate the predictive value of bone and cartilage markers of musculoskeletal injury across two geographically distinct populations of Thoroughbred racehorses, we used the historical data from the study on North American Thoroughbreds completed by one of the authors [21]. Cases from the historical database were selected to match the studied group of Polish racehorses in terms of age, gender, sample collection time (stage of the training season, time from the injury/exit period) and biomarker selection. Selected group involved all 2-year-old racehorses for whom the complete dataset of biomarker measurements was available at three consecutive months leading up to the injury. Control dataset involved three consecutive measurements from racehorses that did not suffer injury in the racing season, obtained in the similar part of the year as the injured group and the Polish population (spring-summer). Therefore, we ensured that all horses enrolled in the analysis were at the similar stage of training during the season of interest. Characterization of both populations is presented in Table 1. This subset of archival data, including 2 year old horses only, has never been analyzed separately before. The recent study uses a different statistical approach for evaluation of predictive value of biomarkers and does not duplicate previously published information.

**Table 1:** Characterization of recent and historical population used in the multivariate predictive models of musculoskeletal injury

Geographical location	Poland	United States (California)
	2 year old	2 year old
Number of horses	26	35
Males	15	18
Females	11	17
Control	18	29
Males		
Females		
Injured	8	6
Males		
Females		
DMD	2	1
Joint injury	3	2
Bone fracture	2	2
Tendonitis	1	1

*DMD* dorsal metacarpal disease

### Statistical analysis

Statistical analysis was performed with a statistical and graphing programs^5^^,.^^6^ A significance level of 0.05 was applied in all analyses. First, serum concentration of each biomarker was averaged over entire study period and compared between the injured and the control group. Data normality was assessed in all groups by Shapiro-Wilk test. Depending on data distribution, means were compared by unpaired t-test or Mann-Whitney test. Next, two-way interaction for the injured and the control group by time period was performed using mixed model ANOVA, with time as within-subject factor and injury as between-subject factor and post-hoc test with Bonferroni correction. Results were presented as mean and standard deviation (SD).

To generate the predictive model, data from the recent and the historical population was normalized and expressed as ratio of the injury/exit sample (Month 0/Month 3, Month 1/Month 3 and Month 2/Month 3 ratio). Therefore, only three first time points were analyzed for each biomarker (as the Month 3 sample served as normalization factor). Normalization of raw values of biomarker concentrations was prerequisite for integration of both databases in one multivariate analysis. The rationale for normalizing data to the last sample, which led to its exclusion from the analysis, is that it would not be useful for assessment of predictive ability of examined biomarkers, as it was collected at the time of injury. The outcome variable was whether or not a horse has been diagnosed with injury during 3 months in the study. Multivariate logistic regression (LOGISTIC procedure^f^) performed on the integrated database was used to classify horses as injured or uninjured (with no regard to the injury type) using separate normalized biomarker levels at each time point. Population origin was entered in the analysis as additional categorical variable. Variables were selected for inclusion using a backward elimination approach. In this approach, the intercept-only model was fit first and results of the Wald test for individual effects were evaluated. The effects with *p* > 0.1 were removed from the model. Next, variables that met the significance level for inclusion were offered to a logistic regression analysis for assessment of their performance in prediction of injury risk. Effect estimates obtained from logistic regression were expressed as odds ratios (OR), and variability as 95% confidence interval (CI).

## Results

Out of 28 horses enrolled in the study, 2 horses left the racetrack before completion of 3 study months due to weak performance unrelated to lameness. Samples from those horses were not included in the analysis. The final population included 26 horses, 15 males and 11 females. Eighteen horses completed the whole study period without manifesting clinical injury, 6 horses were diagnosed with musculoskeletal injury in the third month of the study and 2 horses sustained injury shortly after the end of the study. Due to the close time distance from the last sample collection to injury recognition (one week), these two horses were classified in analysis as injured. Distribution of cases among the trainers was 4 out of 12, 3 out of 10 and 1 out of 6 racehorses in the respective training stables. Injuries were defined as dorsal metacarpal disease (*n* = 2), acute distal limb fracture (n = 2), tendonitis (*n* = 1) and front fetlock joint injury (*n* = 3). Table 1 shows injury distribution in each population.

Data in all compared groups showed normal distribution as evaluated by Shapiro-Wilk test (*p* > 0.05), except of CS846 serum levels in the control group. Nonparametric methods were used for analysis of that biomarker. Looking at serum concentrations of biomarkers averaged over all time points, the injured group demonstrated significantly lower GAG, CPII and OC and higher CTX I levels comparing to control. There was no significant difference in CS846 level between study groups (Table 2).

**Table 2:** Comparison of average serum concentration of biomarkers between control and injured group in Polish racehorse population

Biomarker	Serum concentration (mean ± SD)		*p* value
	Control group	Injured group	
GAG (mcg/mL)	47.29 ± 4.83	42.16 ± 3.34	0.012 *
CS846 (ng/mL)	335.8 ± 126.8	270.6 ± 95.62	0.207
CPII (ng/mL)	3633 ± 590.0	3070 ± 706.7	0.044 *
OC (ng/mL)	59.53 ± 10.07	43.92 ± 5.734	0.0004 **‡**
CTX I (ng/mL)	0.4152 ± 0.13	0.6285 ± 0.08	0.0002 **‡**

* Values differ significantly p < 0.05; ‡ values differ significantly *p* < 0.001. SD – standard deviation

**Fig. 1:**
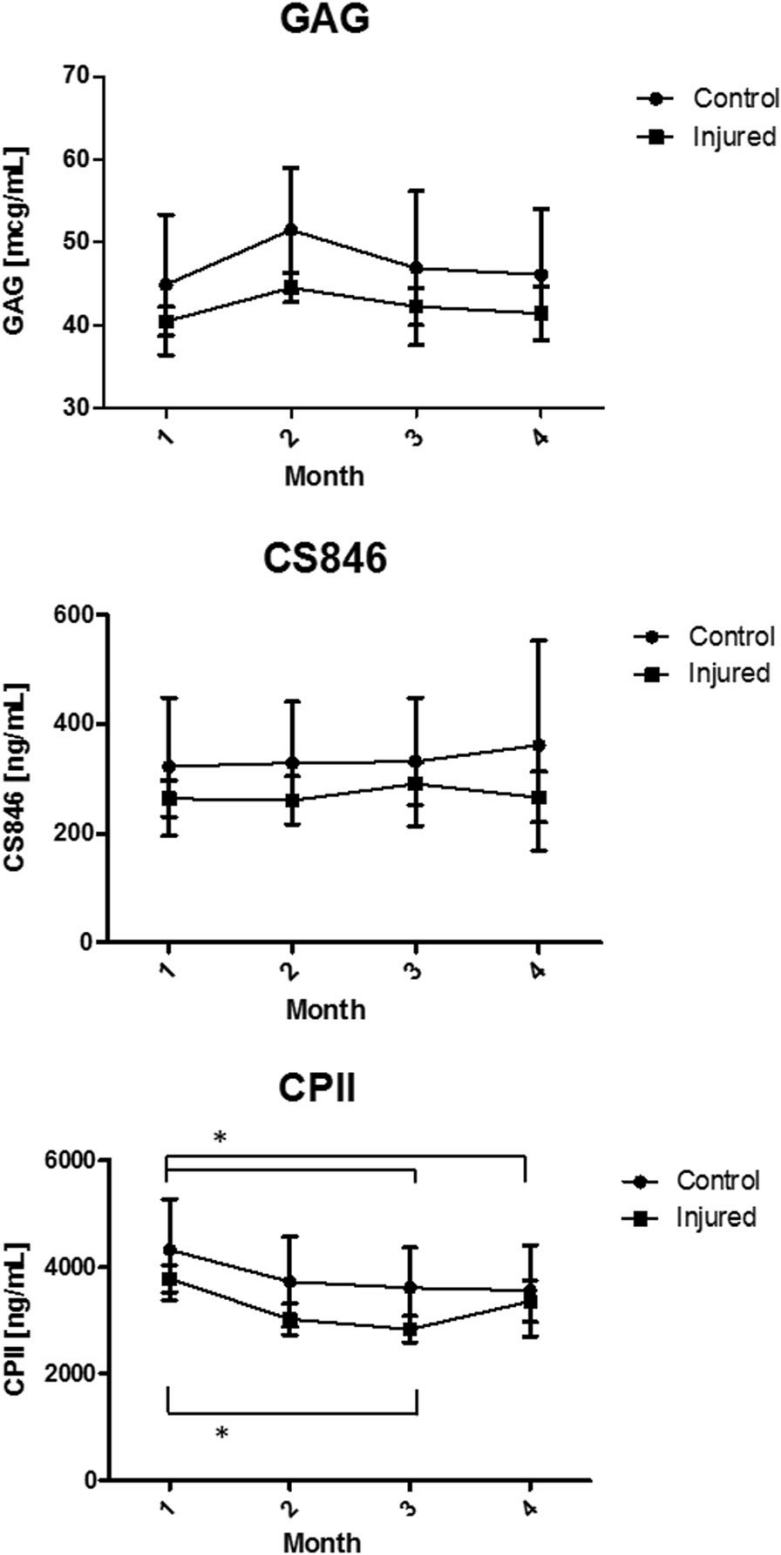
Mean ± SD serum concentration of biomarkers of cartilage metabolism (CPII, GAG and CS846) over 3 months of race training in 2-year-old Thoroughbred horses that finally sustained musculoskeletal injury (*n* = 8) and control horses (*n* = 18). * values differ significantly *p* < 0.05

**Fig. 2:**
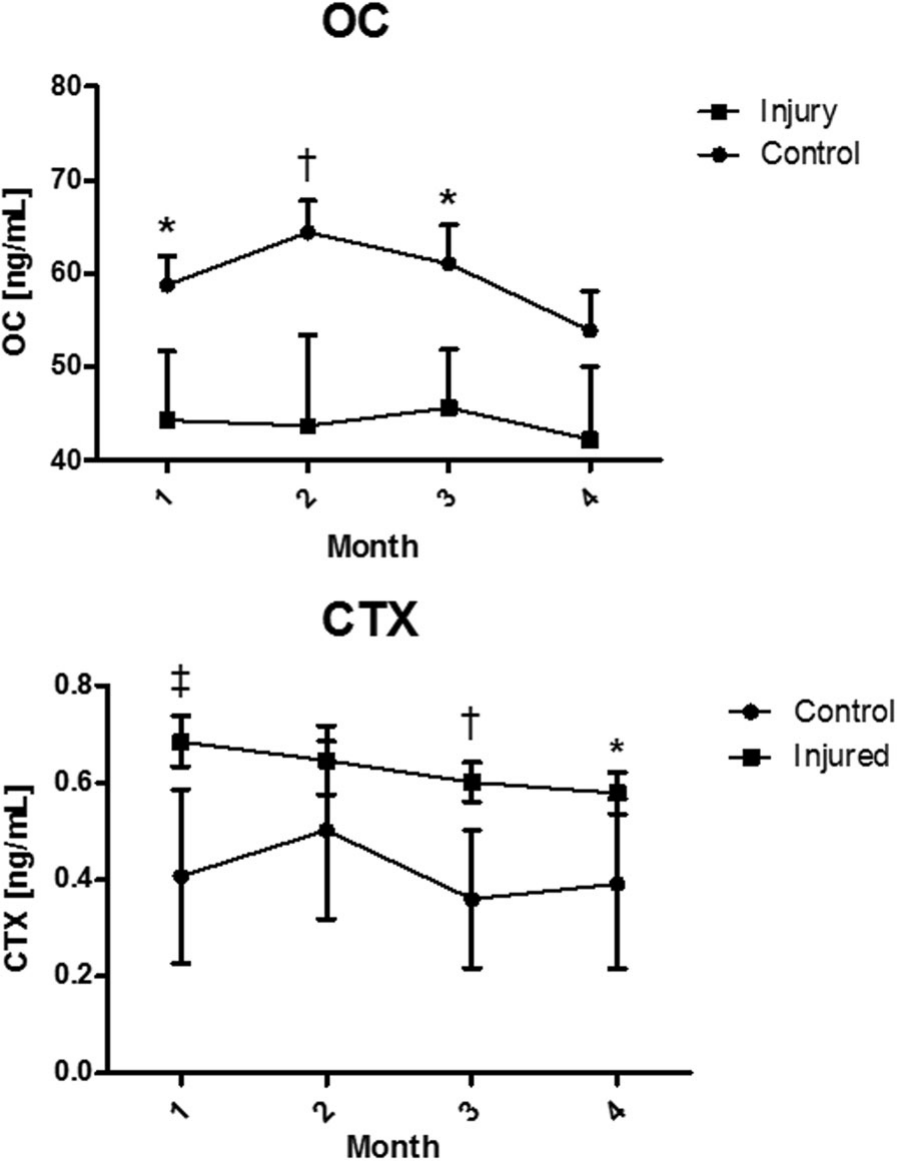
Mean ± SD serum concentration of biomarkers of bone formation (OC) and resorption (CTX I) over 3 months of race training in 2-year-old Thoroughbred horses that finally sustained musculoskeletal injury (n = 8) and control horses (n = 18). * values differ significantly p < 0.05; † values differ significantly *p* < 0.01; ‡ values differ significantly *p* < 0.001

Time did not seem to affect mean biomarker levels in the injured and the control group, with the exception of CPII, where significantly higher concentrations were observed at the baseline point (Fig. 1). Mean OC level in the control group and CTX I in the injured group tended to steadily decrease towards the exit/injury period, although no significant differences were observed (Fig. 2). Comparisons of mean biomarker levels between the injured and the control group at respective time points showed no significant difference for GAG, CS846 and CPII throughout the whole study period (Fig. 1). In the injured group, level of OC was significantly lower than in the control group in all samples except of the endpoint, while CTX I level was significantly higher in baseline, endpoint and Month 2 sample (Fig. 2). In Month 1 sample, CTX I level in the control group approximated that of the injured group, before returning back to baseline in the Month 2 and the endpoint samples.

Sixty-one horses from the current and the historical study were entered into logistic regression analysis, 14 injured and 47 uninjured (see Table 1). Backwards elimination procedure selected CPII level in Month 1 sample and GAG level in Month 2 sample as significant effects for final multivariable logistic regression model (Table 3). These results suggest that there is an association between increased CPII and decreased GAG level and risk of sustaining musculoskeletal injury.

**Table 3:** Results of the logistic regression analysis for samples that demonstrated significant effects in backwards elimination analysis (Wald Chi-Square *p*-value< 0.1)

Biomarker	Time point	Wald Chi-Square Test		Odds Ratio Estimates		
		Wald Chi-Square	p-value	Point Estimate	95% Interval Limits	
CPII	Month 1	3.0435	0.08	1.72	0.94	3.17
GAG	Month 2	5.1169	0.02	0.27	0.09	0.84

## Discussion

According to the current guidelines of OARSI Biomarker Working Group, demonstrating a link between biomarker and clinical outcome in more than one prospective clinical study is fundamental for considering it scientifically sound [43]. This study aimed to reassess usability of the prognostic biomarkers of musculoskeletal disease identified in the North American population of Thoroughbred racehorses [21] in a geographically distinct group of Polish racehorses. The results were expected to reveal how environmental differences may affect biomarker performance as the injury predictor. While direct comparisons of data from both studies were not possible, trends in biomarker levels over time and differences between control and injured group reported in the current study will be contrasted with the previous results from the North American population in the following paragraphs.

The declining trend of OC and CTX I serum levels over time in both control and injured groups (Fig. 2) in the recent study may be interpreted as physiological, age-related change previously described in maturing Thoroughbreds [21, 44]. In the current study, significantly lower OC, and higher CTX I levels were reported in the injured racehorses comparing to the control group. That difference was observed between both values averaged over the whole study period and mean values for the respective time points (Fig. 2). It suggests that balance of bone synthesis and resorption in those individuals was disrupted, leading to loss of bone mechanical resistance and subsequent stress injury. None of these changes were reported in North American racehorses [21], which supports our hypothesis that biomarker profiles of these two populations will differ to some extent, presumably due to varying training environment. Epidemiological studies demonstrated before high rate of skeletal injuries in horses racing on a turf track surface [33, 34]. Indeed, bone injuries were the major group of injuries observed in the Polish racehorses analyzed in this study (Table 1). Although the examined sample cannot be considered representative for population due to its small size, acute disruption in bone turnover equilibrium in the injured horses trained on turf track is an interesting observation that deserves a follow-up study.

In cartilage biomarkers, average serum levels of CPII and GAG were significantly lower in horses that were going to suffer injury compared to control horses (Table 2). The same observation was made in the North American horses that finally sustained DMD (CPII, GAG) or joint injury (GAG), two of the most common injury types diagnosed in the Polish group in the current study and previous studies on the same racehorse population [45]. The lack of significant differences in GAG and CPII levels between groups at the respective time points could have resulted from relatively small size of Polish sample comparing to the previous biomarker study [21]. Glycosaminoglycan is a biomarker of cartilage proteoglycan turnover [46] whose increased serum level can be observed at early stages of osteoarthritis but also in the physiological response of cartilage to exercise [13]. Lower average level of GAG in racehorses that eventually sustained injury as compared to healthy horses may suggest failed structural adaptation of cartilage to the increasing training load. This finding may be supported by a lower average level of CPII in the injured horses, the marker of cartilage-specific type II collagen synthesis that has been shown to increase with exercise [13]. CS846 epitope levels did not reach any significance when analysed at the time of injury or as a part the predictive model, which appears to be in line with recent clinical studies investigating that biomarker for diagnosis of carpal osteochondral lesions [47].

The presented predictive model of injury was based on normalized data derived from the current and the historical study (Table 1). To determine if population origin would affect results of logistic regression analysis, it has been entered into the procedure as an additional categorical variable. However, it was eliminated at the initial stage of the analysis as having no individual effect. That suggested that relation between serum biomarkers and the clinical outcome (presence/absence of injury) is universal across populations and justified using merged databases in the predictive model. This analysis did not reproduce the statistical approach used in the historical study, where all biomarker outcomes were tested together using the support vector machine model. Instead, we aimed to evaluate performance of individual biomarkers at the respective time points in order to determine the most accurate single predictors of injury. Selecting key predictors out of a biomarker panel is essential for their further application in research and practice, as it substantially reduces costs and time of sample analysis. Interestingly, only GAG and CPII, evaluated within two months before the injury were qualified into the model. When compared to the bone markers, GAG has formerly shown the fastest reaction and the highest clinical correlation for detection of experimentally induced equine osteoarthritis [46] and fastest reaction to physiological exercise in healthy horses [13]. Although joint disorders represented only a fourth of all injuries included in the analysis (Table 1), it is possible that articular cartilage metabolism reflects a general response of the skeleton to mechanical overload and products of this reaction can be detected in blood sooner than biomarkers directly related to the structure affected by clinical pathology.

Short reaction time of GAG and CPII may have also contributed to the fact that their predictive value was demonstrated at 1 (GAG) and 2 (CPII) months before injury. In the studies using serum bone markers for injury risk assessment, the average time between sample analysis and injury manifestation was 6 months [19, 20]. Using that time interval, marker of bone resorption, ICTP, was demonstrated to be useful for identification of horses at risk of developing dorsal metacarpal disease [20]. In a longitudinal study of the North American racehorse population, significant changes in OC and CTX I serum levels were observed at 4 to 7 months pre-injury when compared to both injury levels and the control group. Therefore, the time period covered by the current study might have been too short to capture relevant changes in bone marker concentrations. However, presented results suggest that biomarkers of cartilage metabolism may be more useful for detection of racehorses currently at risk of suffering musculoskeletal injury, which could be of practical importance if regular monitoring of GAG and CPII during racing season was performed. CPII and GAG level changes have been previously demonstrated to correlate with imaging and histopathological outcomes in diagnosis and severity classification of equine osteoarthritis [48, 49], further confirming their relevance as biomarkers of joint injury in that species.

One of the potential limitations of the study was application of immunoenzymatic assays for biomarker level measurement that suffered of relatively high intra-assay imprecisions (10,9% in CTX I and 12% in OC ELISA). Due to the comparative nature of the study, we chose to use the same methods that have been previously applied in the North American population. However, further implementation of serum biomarkers in equine medicine could benefit from more precise and preferably fully automated analytic methods.

Although serum biomarkers show promise as a potential screening tool for identification of horses at risk of developing injury, further examination with well recognised diagnostic methods is necessary in order to determine specific pathology type and location. Varied results in bone marker dynamics between distinct racehorse populations suggest that, as an indicator of bone metabolism, serum biochemical marker data should be used in conjunction with clinical examination and diagnostic imaging for more meaningful results.

## Conclusions

This study shows that racehorses in European training system, represented by Polish population sample, might be more prone to disruption of bone turnover homeostasis that is reflected by differences in bone marker serum levels. Environmental factors, particularly training track surface, are proposed as an underlying cause of identified difference in bone metabolism between North American and Polish racehorses. Conversely, cartilage markers appear to show similar dynamics in injured and healthy racehorses regardless of population origin and turn out to be more accurate predictors of approaching injury.

## Additional file

Additional file 1: **Table S1.** Description of training regimen during the study period. (DOCX 12 kb)

## Abbreviations

CPII: c-propeptide of type II procollagen
CS846: chondroitin sulfate epitope
CTX I: c-terminal telopeptide of type I collagen
GAG: total glycosaminoglycans
OARSI: Osteoarthritis Research Society International
OC: osteocalcin

## Footnotes

1 BD Vacutainer blood collection system, Becton, Dickinson and Company, Franklin Lakes, NJ

2 MicroVue Osteocalcin EIA, Quidel Corp., San Diego, CA

3 IBEX Technologies Inc., Montreal, Quebec, Canada

4 Nordic Bioscience Diagnostics, Herlev, Denmark

5 SAS, version 9.2, SAS Institute Inc., Cary, NC

6 Prism 5, GraphPad Software, Inc., La Jolla, CA

## References

[1] Price JS, Jackson B, Eastell R, Wilson AM, Russell RG, Lanyon LE (1995). The response of the skeleton to physical training: a biochemical study in horses. Bone.

[2] Dyson PK, Jackson BF, Pfeiffer DU, Price JS (2008). Days lost from training by two- and three-year-old thoroughbred horses: a survey of seven UK training yards. Equine Vet J.

[3] Olivier A, Nurton JP, Guthrie AJ (1997). An epizoological study of wastage in thoroughbred racehorses in Gauteng, South Africa. J S Afr Vet Assoc.

[4] Cogger N, Evans D, Hodgson D, Reid SW, Perkins N (2008). Incidence rate of musculoskeletal injuries and determinants of time to recovery in young Australian thoroughbred racehorses. Aust Vet J.

[5] Bolwell CF, Rogers CW, French NP, Firth EC (2012). Risk factors for interruptions to training occurring before the first trial start of 2-year-old thoroughbred racehorses. N Z Vet J.

[6] Johnson BJ, Stover SM, Daft BM, Kinde H, Read DH, Barr BC (1994). Causes of death in racehorses over a 2 year period. Equine Vet J.

[7] Estberg L, Stover SM, Gardner IA, Johnson BJ, Jack RA, Case JT (1998). Relationship between race start characteristics and risk of catastrophic injury in thoroughbreds: 78 cases (1992). J Am Vet Med Assoc.

[8] Beisser AL, McClure S, Wang C, Soring K, Garrison R, Peckham B (2011). Evaluation of catastrophic musculoskeletal injuries in thoroughbreds and quarter horses at three midwestern racetracks. J Am Vet Med Assoc.

[9] Lejeune JP, Serteyn D, Gangl M, Schneider N, Deby-Dupont G, Deberg M (2007). Plasma concentrations of a type II collagen-derived peptide and its nitrated form in growing ardenner sound horses and in horses suffering from juvenile digital degenerative osteoarthropathy. Vet Res Commun.

[10] Misumi K, Vilim V, Hatazoe T, Murata T, Fujiki M, Oka T (2002). Serum level of cartilage oligomeric matrix protein (COMP) in equine osteoarthritis. Equine Vet J.

[11] Gangl M, Serteyn D, Lejeune JP, Schneider N, Grulke S, Peters F (2007). A type II-collagen derived peptide and its nitrated form as new markers of inflammation and cartilage degradation in equine osteochondral lesions. Res Vet Sci.

[12] Jackson BE, Smith RK, Price JS (2003). A molecular marker of type I collagen metabolism reflects changes in connective tissue remodelling associated with injury to the equine superficial digital flexor tendon. Equine Vet J.

[13] Frisbie DD, Al-Sobayil F, Billinghurst RC, Kawcak CE, McIlwraith CW (2008). Changes in synovial fluid and serum biomarkers with exercise and early osteoarthritis in horses. Osteoarthr Cartil.

[14] McIlwraith CW, Frisbie DD, Rodkey WG, Kisiday JD, Werpy NM, Kawcak CE (2011). Evaluation of intra-articular mesenchymal stem cells to augment healing of microfractured chondral defects. Arthroscopy.

[15] Frisbie DD, Ray CS, Ionescu M, Poole AR, Chapman PL, McIlwraith CW (1999). Measurement of synovial fluid and serum concentrations of the 846 epitope of chondroitin sulfate and of carboxy propeptides of type II procollagen for diagnosis of osteochondral fragmentation in horses. Am J Vet Res.

[16] McIlwraith CW (2005). Use of synovial fluid and serum biomarkers in equine bone and joint disease: a review. Equine Vet J.

[17] Verwilghen D, Busoni V, Gangl M, Franck T, Lejeune JP, Vanderheyden L (2009). Relationship between biochemical markers and radiographic scores in the evaluation of the osteoarticular status of warmblood stallions. Res Vet Sci.

[18] Nicholson AM, Trumble TN, Merritt KA, Brown MP (2010). Associations of horse age, joint type, and osteochondral injury with serum and synovial fluid concentrations of type II collagen biomarkers in thoroughbreds. Am J Vet Res.

[19] Jackson BF, Dyson PK, Lonnell C, Verheyen KLP, Pfeiffer DU, Price JS (2009). Bone biomarkers and risk of fracture in two- and three-year-old thoroughbreds. Equine Vet J.

[20] Jackson BF, Lonnell C, Verheyen KLP, Dyson P, Pfeiffer DU, Price JS (2005). Biochemical markers of bone metabolism and risk of dorsal metacarpal disease in 2-year-old thoroughbreds. Equine Vet J.

[21] Frisbie DD, McIlwraith CW, Arthur RM, Blea J, Baker VA, Billinghurst RC (2010). Serum biomarker levels for musculoskeletal disease in two- and three-year-old racing thoroughbred horses: a prospective study of 130 horses. Equine Vet J.

[22] Vervuert I, Coenen M, Wedemeyer U, Harmeyer J (2002). Biochemical markers of bone activity in young standardbred horses during different types of exercise and training. J Vet Med A Physiol Pathol Clin Med.

[23] Inoue Y, Matsui A, Asai Y, Aoki F, Yoshimoto K, Matsui T (2008). Response of biochemical markers of bone metabolism to exercise intensity in thoroughbred horses. J Equine Sci.

[24] Hiney KM, Potter GD, Gibbs PG, Bloomfield SM (2000). Response of serum biochemical markers of bone metabolism to training in the juvenile racehorse. J Equine Vet Sci.

[25] Ramzan PHL, Palmer L (2011). Musculoskeletal injuries in thoroughbred racehorses: a study of three large training yards in Newmarket, UK (2005-2007). Vet J.

[26] Parkin TDH (2008). Epidemiology of racetrack injuries in racehorses. Vet Clin North Am - Equine Pract.

[27] Perkins NR, Reid SW, Morris RS (2005). Risk factors for musculoskeletal injuries of the lower limbs in thoroughbred racehorses in New Zealand. N Z Vet J.

[28] Verheyen K, Price J, Lanyon L, Wood J (2006). Exercise distance and speed affect the risk of fracture in racehorses. Bone.

[29] Hernandez J, Hawkins DL, Scollay MC (2001). Race-start characteristics and risk of catastrophic musculoskeletal injury in thoroughbred racehorses. J Am Vet Med Assoc.

[30] Boston RC, Nunamaker DM (2000). Gait and speed as exercise components of risk factors associated with onset of fatigue injury of the third metacarpal bone in 2-year-old thoroughbred racehorses. Am J Vet Res.

[31] Oikawa M, Kusunose R (2005). Fractures sustained by racehorses in Japan during flat racing with special reference to track condition and racing time. Vet J.

[32] Parkin TDH, Clegg PD, French NP, Proudman CJ, Riggs CM (2005). Risk factors for fatal lateral condylar fracture of the third metacarpus/metatarsus in UK racing. Equine Vet J.

[33] Verheyen K, Price J, Wood J. Associations between training surfaces and risk of fracture in thoroughbred racehorses in training. Proceedings of the 11th International Symposium on Veterinary Epidemiology and Economics. 2006. http://www.sciquest.org.nz/node/63891.

[34] Mohammed HO, Hill T, Lowe J (1991). Risk factors associated with injuries in thoroughbred horses. Equine Vet J.

[35] Hill AE, Stover SM, Gardner IA, Kane AJ, Whitcomb MB (2001). Risk factors for and outcomes of noncatastrophic suspensory apparatus injury in thoroughbred racehorses. J Am Vet Med Assoc.

[36] Anthenill LA, Stover SM, Gardner IA, Hill AE (2007). Risk factors for proximal sesamoid bone fractures associated with exercise history and horseshoe characteristics in thoroughbred racehorses. Am J Vet Res.

[37] Ross MW, Dyson SJ, Ross MW, Dyson SJ (2011). The European thoroughbred. Diagnosis and Management of Lameness in the horse.

[38] Arthur RM, Ross MW, Moloney PJ, Cheney MW, Ross MW, Dyson SJ (2011). North American thoroughbred. Diagnosis and Management of Lameness in the horse.

[39] Mitchell A, Wright G, Samson SN, Martin M, Cummings K, Gaddy D, et al. Clodronate improves lameness in horses without changing bone turnover markers. Equine Vet J. 2018. 10.1111/evj.13011. 10.1111/evj.13011 30153345

[40] Hoyt S, Siciliano PD (1999). A comparison of ELISA and RIA techniques for the detection of serum osteocalcin in horses. Proc Equine Nutr Physiol Symp.

[41] Billinghurst RC, Brama PAJ, van Weeren PR, Knowlton MS, McIlwraith CW (2003). Significant exercise-related changes in the serum levels of two biomarkers of collagen metabolism in young horses. Osteoarthr Cartil.

[42] Farndale RW, Buttle DJ, Barrett AJ (1986). Improved quantitation and discrimination of sulphated glycosaminoglycans by use of dimethylmethylene blue. Biochim Biophys Acta.

[43] Kraus VB, Burnett B, Coindreau J, Cottrell S, Eyre D, Gendreau M (2011). Application of biomarkers in the development of drugs intended for the treatment of osteoarthritis. Osteoarthr Cartil.

[44] Price JS, Jackson BF, Gray JA, Harris PA, Wright IM, Pfeiffer DU (2001). Biochemical markers of bone metabolism in growing thoroughbreds: a longitudinal study. Res Vet Sci.

[45] Turlo A, Cywinska A, Czopowicz M, Witkowski L, Niedzwiedz A, Slowikowska M (2015). The effect of different types of musculoskeletal injuries on blood concentrations of serum amyloid a in thoroughbred racehorses. PLoS One.

[46] Frisbie D, Al-Sobayil F, Billinghurst R, McIlwraith CW. Serum markers differentiate exercise from pathology and correlate to clinical parameters of pain in an osteoarthritic model. New Orleans: Transactions of the 49th Annual Meeting of the Orthopedic Research Society; 2003. https://www.ors.org/Transactions/49/0751.pdf.

[47] Hipolito C, Hugo F, Oscar A, Benjamin U, Gabriel M (2016). Concentration of the CS-846 epitope in serum and synovial fluid of horses with different grades of osteochondral fragments in the carpal joints. Gen Med.

[48] Legrand CB, Lambert CJ, Comblain FV, Sanchez C, Henrotin YE (2017). Review of soluble biomarkers of osteoarthritis: lessons from animal models. Cartilage.

[49] Coppelman E. The use of biomarkers to determine the severity of osteoarthritis in the tarsus of an older horse. Population. 2017; https://conservancy.umn.edu/bitstream/handle/11299/194660/Coppelman_umn_0130M_18905.pdf?sequence=1. Accessed 12 Dec 2018.

