# Supplementary material for: Revisiting predictive biomarkers of musculoskeletal injury in thoroughbred racehorses: longitudinal study in polish population
Source: BMC Vet Res. 2019 Feb 26;15:66. doi: 10.1186/s12917-019-1799-7 (PMC6390350; doi:10.1186/s12917-019-1799-7)
Supplement: Supplementary file 1 — Table S1. Description of training regimen during the study period. (DOCX 12 kb) [file 12917_2019_1799_MOESM1_ESM.docx]

**Table S1.** Description of training regimen during the study period.

| **Month** | **Workout** | **Description** |
| --- | --- | --- |
| April-May | canter | 1000-1400m workouts at the speed ≈ 500 m/min. Gradually expanding canter distance |
| June | canter  introducing gallop* | Increasing speed on the last 400 m of 1400 m canter  Gradually expanding gallop distance |
| end of June | canter  gallop | 2 gallops within 7-10 day period with 800 m at the speed ≈ 900 m/min. |
| > July | first 2-years-old races | Racing distance 1200 m |

* Exact time of introducing high-speed workouts differed between trainers and individual horses.
